# Fragmentation and Coverage Variation in Viral Metagenome Assemblies, and Their Effect in Diversity Calculations

**DOI:** 10.3389/fbioe.2015.00141

**Published:** 2015-09-17

**Authors:** Rodrigo García-López, Jorge Francisco Vázquez-Castellanos, Andrés Moya

**Affiliations:** ^1^Área de Genómica y Salud, Fundación para el Fomento de la Investigación Sanitaria y Biomédica de la Comunidad Valenciana (FISABIO)-Salud Pública, Valencia, Spain; ^2^Institut Cavanilles de Biodiversitat i Biologia Evolutiva, Universitat de València, Paterna, Spain; ^3^Consorcio de Investigación Biomédica en Red especializado en Epidemiología y Salud Pública (CIBERESP), Madrid, Spain

**Keywords:** viral metagenomics, assembler, OTU, diversity

## Abstract

Metagenomic libraries consist of DNA fragments from diverse species, with varying genome size and abundance. High-throughput sequencing platforms produce large volumes of reads from these libraries, which may be assembled into contigs, ideally resembling the original larger genomic sequences. The uneven species distribution, along with the stochasticity in sample processing and sequencing bias, impacts the success of accurate sequence assembly. Several assemblers enable the processing of viral metagenomic data *de novo*, generally using overlap layout consensus or de Bruijn graph approaches for contig assembly. The success of viral genomic reconstruction in these datasets is limited by the degree of fragmentation of each genome in the sample, which is dependent on the sequencing effort and the genome length. Depending on ecological, biological, or procedural biases, some fragments have a higher prevalence, or coverage, in the assembly. However, assemblers must face challenges, such as the formation of chimerical structures and intra-species variability. Diversity calculation relies on the classification of the sequences that comprise a metagenomic dataset. Whenever the corresponding genomic and taxonomic information is available, contigs matching the same species can be classified accordingly and the coverage of its genome can be calculated for that species. This may be used to compare populations by estimating abundance and assessing species distribution from this data. Nevertheless, the coverage does not take into account the degree of fragmentation, or else genome completeness, and is not necessarily representative of actual species distribution in the samples. Furthermore, undetermined sequences are abundant in viral metagenomic datasets, resulting in several independent contigs that cannot be assigned by homology or genomic information. These may only be classified as different operational taxonomic units (OTUs), sometimes remaining inadvisably unrelated. Thus, calculations using contigs as different OTUs ultimately overestimate diversity when compared to diversity calculated from species coverage. In order to compare the effect of coverage and fragmentation, we generated three sets of simulated Illumina paired-end reads with different sequencing depths. We compared different assemblies performed with RayMeta, CLC Assembly Cell, MEGAHIT, SPAdes, Meta-IDBA, SOAPdenovo, Velvet, Metavelvet, and MIRA with the best attainable assemblies for each dataset (formed by arranging data using known genome coordinates) by calculating different assembly statistics. A new fragmentation score was included to estimate the degree of genome fragmentation of each taxon and adjust the coverage accordingly. The abundance in the metagenome was compared by bootstrapping the assembly data and hierarchically clustering them with the best possible assembly. Additionally, richness and diversity indexes were calculated for all the resulting assemblies and were assessed under two distributions: contigs as independent OTUs and sequences classified by species. Finally, we search for the strongest correlations between the diversity indexes and the different assembly statistics. Although fragmentation was dependent of genome coverage, it was not as heavily influenced by the assembler. The sequencing depth was the predominant attractor that influenced the success of the assemblies. The coverage increased notoriously in larger datasets, whereas fragmentation values remained lower and unsaturated. While still far from obtaining the ideal assemblies, the RayMeta, SPAdes, and the CLC assemblers managed to build the most accurate contigs with larger datasets while Meta-IDBA showed a good performance with the medium-sized dataset, even after the adjusted coverage was calculated. Their resulting assemblies showed the highest coverage scores and the lowest fragmentation values. Alpha diversity calculated from contigs as OTUs resulted in significantly higher values for all assemblies when compared with actual species distribution, showing an overestimation due to the increased predicted abundance. Conversely, using PHACCS resulted in lower values for all assemblers. Different association methods (random-forest, generalized linear models, and the Spearman correlation index) support the number of contigs, the coverage, and fragmentation as the assembly parameters that most affect the estimation of the alpha diversity. Coverage calculations may provide an insight into relative completeness of a genome but they overlook missing fragments or overly separated sequences in a genome. The assembly of a highly fragmented genomes with high coverage may still lead to the clustering of different OTUs that are actually different fragments of a genome. Thus, it proves useful to penalize coverage with a fragmentation score. Using contigs for calculating alpha diversity result in overestimation but it is usually the only approach available. Still, it is enough for sample comparison. The best approach may be determined by choosing the assembler that better fits the sequencing depth and adjusting the parameters for longer accurate contigs whenever possible whereas diversity may be calculated considering taxonomical and genomic information if available.

## Introduction

The human body hosts more than 3000 species of bacteria, accounting for 10^14^ cells, ~10-fold the total number of human cells in healthy human adults (Bäckhed et al., 2005). On the other hand, viruses are a broad group of agents that depend on host cells to replicate their genetic material and assemble themselves. They are far more abundant than any living organism, a`ccounting for more than 10^31^ particles in the planet (Minot et al., 2011) and it has been estimated that there may be potentially more than 320,000 different viruses in mammals (Anthony et al., 2013). Furthermore, the viral community plays an important role in the establishment shaping of their bacterial counterpart (Abeles and Pride, 2014). Although the relationship between both communities remains obscure, it is a field of growing interest.

The past decade has seen the advent and progress of meta-omic sciences, reflected by international collaborative efforts toward the understanding of this collection of microorganisms and their role in different human health-related conditions, such as the Human Microbiome Project and the MetaHIT (NIH HMP Working Group et al., 2009; Qin et al., 2010). Current generation sequencing has enabled metagenomic studies of inclusive spectra of microorganisms in different environmental niches within the human body. Studies of the viral populations in humans have found smaller variation in viral diversity than in their bacterial counterparts, showing a low overall diversity and highly variable viral profiles for the same niche over time, whereas variation between different individuals was higher (Wen et al., 2008; Pérez-Brocal et al., 2013). In twins and mother studies, it was reported that bacteriophages distributions were independent of kinship (Reyes et al., 2010). Still, fairly few studies have focused entirely on viral metagenomics (viromics). This is partly because of the challenging and non-standardized endeavor of obtaining and isolating viral particles from samples, as well as the limited number of species that have been characterized. Furthermore, taxonomic classification is not consistent, with some sequences bearing taxonomic labels based solely on a common gene or partial sequence, while many lack the higher taxonomic ranks (order, family) (Lauber and Gorbalenya, 2012). Viral metagenome assemblies are further complicated by chimeric contig formation, although viral–bacterial chimeras are far less common (negligible) than viral-only chimeras that may arise from horizontal transfer or over-represented functions in the metagenome related to viral replication (Vázquez-Castellanos et al., 2014).

Most taxonomic studies in bacteria rely in the existence of the 16S rRNA gene, a universal marker conserved throughout the bacterial domain. Hypervariable regions of these sequences allow for the classification of bacteria with a species level resolution in most cases. No analogous universal molecular marker exists in viruses and taxonomic analyses are actually done with metagenomics (Rosario and Breitbart., 2011). Each contig or fragment is compared to extant sequences in viral databases, usually employing some method of sequence alignment and homology comparison for taxonomic assignment, or is classified according to genomic information by reference-free methods. There are several challenges in the study of viral metagenomes, which can be grouped according to the type of limitation they represent. Most can be summarized in three categories: method related, genomic content related, and database related. For the first group, the lack of protocol standardization limits the extent to which results may be compared. Some research groups separate fractions with cesium chloride gradients prior to extractions (Robles-Sikisaka et al., 2013), while others use mechanical disruption and filters (Pérez-Brocal et al., 2013). Most use DNAse/RNAse cocktails in order to remove contaminant DNA prior to lysate the viral envelopes containing the nucleic acids (Pérez-Brocal et al., 2013; Robles-Sikisaka et al., 2013). Extraction methods are also varied but have been simplified by the availability of kits.

The success in obtaining a virome (an environmental viral metagenome) depends heavily on the quantity and quality of the nucleic acids. Metagenomic studies are commonly aided by sequence-independent amplification that aims to enrich samples. Some of the most common are multiple displacement amplification and sequence-independent single primer amplification (SISPA) but biases have been reported (Zou et al., 2003; Kim and Bae, 2011; Marine et al., 2014). Also, the bioinformatic approach that is used to process the resulting sequences is critical. The second type of limitation depends on the nucleic acid content in the sample and is influenced by the ecology of the sample and the replication mechanism of each virus. Abundance is differential and is heavily influenced by the actual viral content in the niche that is studied. Furthermore, viral metagenomes are far from stable but tend to have a fairly constant diversity at higher taxonomic levels in the same individual while having a high variability among inter-individual samples (Wen et al., 2008). Unless an infected area is drawn during sample, most homology-assigned viral species in a virome tend to match bacteriophage sequences. The viral load of most other viruses may be too low to be represented in a sample and only those in a replicating phase may have enough copies for a metagenomic survey to actually gather enough information for genomic assembly. Thus, distribution is of utmost relevance in viral metagenomics. The final limitation in viromics is the scarce availability of reliable repositories of viral sequences (Rosario and Breitbart., 2011). The INSD collaborative effort has the largest collection of such sequences. Other databases focus on specific viruses, mostly well-characterized pathogens, or genes with clinical relevance. However, in viral metagenomic samples, the majority of the sequences come from unknown or poorly characterized viruses. Although databases continue to grow, most novel viruses have only *in silico* predicted open reading frames (ORFs) or incomplete sequences available. Most bioinformatic tools for taxonomic classification rely on sequence alignments to determine homology to a target sequence. With no template sequence (reference genome) to compare against in most cases, a highly rate of horizontal transfer and heavily conserved functions in viruses (transposases, etc.), there is usually no way of identifying most of the sequences (Dutilh et al., 2014). In viral metagenomics, a significant proportion of the sequences share very low similarity to known genomes. In the absence of homologous sequences, reference-free classification methods may be used for binning these fragments according to potential phylogenetic relationships. These depend on genomic-grade differences, such as CG content and k-mer distribution or oligonucleotide frequency. Different genes or genomes display specific patterns and are alignment-independent, allowing for a distance calculation and comparison of all fragments based on frequencies alone (Trifonov and Rabadan, 2010). However, the extent of success in viral metagenomics is complicated due to horizontal gene transfer and uneven distribution of the genomes, reflected in low specificity and sensibility, with a high numbers of false positives (Vázquez-Castellanos et al., 2014). On the other hand, reference-free methods are powerful methods for filtering bacterial sequences in viral metagenomes.

Current generation sequencers stand out for having a high throughput (e.g., Illumina outputs are commonly in the Gbp range). This enables a fairly exhaustive exploration of the genomic content of all the species found in viromic studies. However, genome completeness depends on the species’ abundance and normally, no genome is recovered entirely, even for most prevalent species. The resulting genome fragmentation is therefore highly variable between species and depends heavily on their distribution in the sample and the sequencing effort, which is the total number of sequences generated per sample. The fragmentation is not commonly estimated in viral metagenomic studies but it is an inherent problem that highlights the importance of assemblers, algorithms that are commonly used to achieve *in silico* reconstructions of longer sequences, contigs, or scaffolds.

Most assemblers were formerly designed for gene and genomic assemblies, focusing on the reconstruction of a single long sequence with an overall same coverage. However, metagenomics is challenging as it requires reconstructing several long DNA fragments belonging to different species with variable genome sizes and distribution in the samples. To address this, some metagenomic assemblers and specific implementations of existent ones have appeared in recent years (Peng et al., 2011; Boisvert et al., 2012; Namiki et al., 2012). Assemblers build longer sequences which, in the case of viral metagenomics, ideally represent the reconstructed fragments of a virus’ genome that may be present in a sample. Assembled contigs vary depending on the underlying abundance of the population and are supported by the assembly statistics. Generally, greater sequencing depths produce larger and better supported contigs.

As we assessed in a pervious study (Vázquez-Castellanos et al., 2014), functional annotation may be assisted by the implementation of an assembling step. This is especially true for short sequences as an accurate classification of sequences is more complicated for shorter reads. Current upgrades to the Illumina platform (V3 sequencing kit) produce 2 bp × 300 bp paired-end reads, resulting in a theoretical ~550–600 bp long reads in a MiSeq desktop sequencer producing up to 50 million (M) reads per flow cell.

Assemblers use different approaches to generate larger sequences from the reads but they can be classified into two main types: overlap layout consensus (OLC) and de Bruijn assemblers (Li et al., 2010). OLC assemblers such as MIRA (Chevreux et al., 1999) rely on the alignment and detection of overlapping of reads and have been especially important for sequencing platforms producing longer reads, such as 454. De Bruijn assemblers, such as CLC (CLC Bio, 2012), Meta-IDBA (Peng et al., 2011), SOAPdenovo (Li et al., 2012), RayMeta (Boisvert et al., 2012), Velvet (Zerbino and Birney, 2008), and Metavelvet (Namiki et al., 2012) split sequences into smaller word size fragments that can be indexed, allowing for the assembly of shorter reads. The de Bruijn graphs approach has proven useful to handle larger data volumes and the short-length reads of Illumina platforms (Li et al., 2012). Recent developments have implemented new approaches on de Bruijn graphs, such as the succinct de Bruijn graphs of MEGAHIT (Li et al., 2015) and SPAdes’ paired and multi-sized de Bruijn graphs (Bankevich et al., 2012).

In the resulting metagenomic assemblies, there is also variation within contigs. As some segments are more prone to be sequenced than others, due to ecological, biological, or procedural biases, a differential spectrum is generated for each contig. The times a given fragment in a genome is present in an assembled sequence is referred to as the coverage. In metagenomics, mean coverages are estimators that can be used for assessing fragment diversity in a sample. They are calculated for each contig as the total sum of nucleotides from all fragments forming the contig, divided by the actual length of the resulting assembled sequence. Whenever taxonomic and genomic information is available for a set of contigs, a species’ mean coverage may be estimated instead, by calculating the total bases of all contigs and unassembled sequences belonging to that species divided by the total length of its genome.

The diversity calculated from the species coverage is more accurate from an ecological point of view than contig coverage but this information is not available in all cases. Minority species in a sample are particularly highly fragmented and only small contigs are built from their surveyed DNA. In the case of taxonomically undetermined species, these remain unrelated to one another, even if they actually belong to the same species. Reference-free classifications may help but they have little specificity and sensibility in viral metagenomes (Vázquez-Castellanos et al., 2014). In such cases, contig coverage may be used to evaluate their presence in a sample by considering contigs as operational taxonomic units (OTUs). Differences between samples can be assessed with this but the approach has some disadvantages. Although the coverage can provide an insight into contig distribution, it may not be representative of species distribution and may lead to diversity overestimation as more different taxa are seemingly present. Several non-homologous contigs may belong to a single species but they are considered different OTUs under this approach.

In this work, we assessed the success of different assemblers in building accurate contigs from viral metagenomic data using simulated data with different sequencing depths. Assembly statistics are provided, including a proposed genome fragmentation index, for comparison against the best attainable assembly of each set. The fragmentation score was used to calculate and compare a penalized coverage that depends on the genome completeness. Additionally, diversity was analyzed using mean contig coverage (OTUs as contigs) and mean species coverage (OTUs as GIs) in order to evaluate the difference between both approaches. Finally, the effect of different statistics over the assemblies was determined to detect which are the most influential.

The assembling step is of utmost importance in viral metagenomics as taxonomy is commonly inferred from sequence alignments with sequences coming from known virus databases. The large unknown fraction is usually impossible to assign to existing records, but appear consistently in virus metagenomic studies (Dutilh et al., 2014). OTUs drawn from contigs and fragment abundance as mean contig coverage help in comparing samples when no reference is available but overestimate actual diversity within them. To this end, we assessed the effect of different types of assembly and their statistics on different estimators of alpha diversity.

## Materials and Methods

### General strategy overview

Simulated datasets bearing three different sequencing depths were generated. These were then assembled using nine different programs. An ideal assembly was generated from reads’ coordinates as the predicted best achievable contig dataset having no chimeras and the longest fragments. Assembly statistics were calculated for all, including the ideal assemblies.

Taxonomy was assigned to fragments comprising the resulting assemblies by mapping them to the reference genomes. The correlations between the different sample statistics were assessed. The assemblies were analyzed via dimensional reduction to compare them with the ideal assemblies. The most influential variables (assembly statistics) across datasets were identified. Hierarchical clusters were formed to group the different assemblies.

The mean coverage and fragmentation were calculated for each species and for each whole dataset. Using the coverage and fragmentation profiles, a distance was calculated from the assemblies to the ideal assembly to compare them and evaluate their effect in the success of assembling an accurate viral metagenome. An additional penalized-coverage profile was calculated and analyzed by integrating the fragmentation score.

In order to evaluate the effect on the alpha diversity estimators of using separate contigs, rather defined species (as occurs with undetermined species) in the calculation, we estimated different diversity and richness indexes for each assembly. Different association methods were employed to evaluate which statistics had the greater impact on the calculated diversity.

### Simulated datasets

Sets of Illumina MiSeq desktop sequencer 2 bp × 300 bp paired-end reads were simulated using modified versions of the scripts from the metagenomic sequence simulator “Better Emulation for Artificial Reads” (BEAR) (Johnson et al., 2014). Data generation was not straightforward since scripts required some changes in order to work properly.

Error rate and quality error models were first generated using the algorithm “duplicate read inferred sequencing error estimation” (DRISEE) (Keegan et al., 2012) with real paired-end (2 bp × 300 bp) sequencing output (fastq) from an Illumina MiSeq from Nextera metagenomic libraries (FISABIO, unpublished data). Since it was reported that DRISEE tends to overestimate errors in metagenomic data due to the presence of conserved artificial sequences, such as adapters (Eren et al., 2014), we removed such sequences using the Cutadapt algorithm.

Relative abundance data were extracted from a virome that was obtained from the study by Reyes et al. (2010) and analyzed in a previous work (Vázquez-Castellanos et al., 2014) and the corresponding genome sequences were downloaded from the NCBI RefSeq database. Paired-end simulations were carried out with BEAR using the same abundance values reported in the actual data, which contained 578 different species that had complete genomes available, as well as the models created with DRISEE. For additional details on the methods, please refer to Presentation S1 in Supplementary Material.

Three 2 × 300 datasets were generated, emulating different throughput scenarios for an Illumina MiSeq using the V3 sequencing kit: 1.5 Gbp, 150, and 15 Mbp, corresponding to 5, 0.5, and 0.05M reads, respectively, the expected optimal throughputs for a flow cell with 10, 100, and 1000 samples. Hereafter, our datasets were called 5, 0.5, and 0.05M. The abundance is reported in Table S1 in Supplementary Material.

For all datasets, the resulting datasets were analyzed with FastQC and PRINSEQ (Schmieder and Edwards, 2011) to identify low accuracy base calling, low complexity data, non-IUPAC characters, and k-mer presence. Low quality bases were trimmed and problematic sequences were removed. Only those having a pair were considered for the rest of the analysis (singletons were removed).

As the sequences had traceable information relating the corresponding reference genome used to generate them, the abundance of each species was calculated for every dataset. A chi squared test was carried out to verify that all datasets actually matched the distribution of the original distribution of the data used to simulate them.

### Assemblers

Nine assemblers were selected to process the simulated data: CLC, IDBA, MEGAHIT, MIRA, RayMeta, SOAPdenovo, SPAdes, Velvet, and Metavelvet. While MIRA is an OLC assembler, the rest use de Bruijn graph approaches with some variations (SPAdes uses modified paired and multi-sized de Bruijn graphs and MEGAHIT uses succinct de Bruijn graphs). MEGAHIT, Metavelvet, Meta-IDBA, and RayMeta were implementations originally designed to address the assembly of metagenomic data. All datasets were inputted as paired-end data in all assemblers.

The k-mer was set to optimize the N50 statistic in the assemblies (a rough measure of the size of the assembly). When the summation of total base pairs in a dataset is calculated and sequences are sorted by length, this value corresponds to the sequence length that halves the cumulative total base pairs.

Assemblies of all three datasets were done using 31 bp k-mers with Ray, 47 for Velvet and Metavelvet and 91 for SOAPdenovo. The Meta-IDBA assembler and MEGAHIT use a series of k-mer iterations that were set to 10–100 for Meta-IDBA and 21–125 and 25–125 for the low and high-covered metagenomes for MEGAHIT. Both assemblers used a step of 10 between iterations. The CLC assembler uses different k-mers that are determined by the total amount of input nucleotides, thus using a k-mer of 19 for the 0.05M dataset, k21 for the 0.5M dataset, and 23 for the larger 5M set. The SPAdes assembler used a vector of k-mers to iterate its assembly, which was set to 21, 33, 55, 77, 99, and 127. MIRA was set to an overlap of 25, a default parameter for Illumina data.

All assemblers produced a single contig fasta file containing the resulting assembly for each dataset. These files were mapped using Bowtie2 (Langmead and Salzberg, 2012) against their original reads to identify those that were now forming contigs. In-house Perl scripts were programed to calculate assembly metrics. The different assembly statistics calculated for each contig spectrum in order to assess the performance of each assembly are shown in Table S2 in Supplementary Material. They describe the amount of reads assembled into contigs (%reads_assembled), the average coverage per contig(Mean_contig_coverage), the number of miss-assembled contigs that resulted in chimeric sequences in different taxonomic levels (%Chim_gi,%Chim_Species,%Chim_genus,%Chim_family and the%Chim_order), the overall length and coverage estimators per assembly (N50, Largest_contig and the Mean_contig_coverage), and the fragmentation of the present genomes (Mean_Fragmentation, Num_contig and the Mean_contig_coverage). The mean and SD of each statistic was calculated for every assembler and for the different datasets to compare them by method and sequencing depth respectively (Tables S3 and S4 in Supplementary Material).

A fragmentation (or completeness) score was calculated as the total summation of fragment length to genome length ratios divided by the total number of fragments per contig. This gives a score that goes from 0 to 1 with the latter being the optimal score. The mean fragmentation per assembly was calculated as the summation of all genome fragmentations divided by the total number of different genomes distinguished by a unique GenInfo Identifier (NCBI’s GIs) on each simulation.

Chimeric contigs were identified using the taxonomic information of the original genomes that were used for simulations. This was calculated per GI and per taxonomic ranks (species, genus, family, and order). Contigs composed of at least 90% reads belonging to a single taxon were labeled chimera-free, whereas the rest (those having more than 10% reads assigned to alternative taxa) added up toward a chimeric contig percentage, which was calculated for each assembly. This was set to avoid marking large contigs with high coverage as bad just for a partially inaccurate assembly.

Taxonomy in viruses is missing rank labels, mainly in the order and genus levels. To properly identify chimeric contigs undetermined ranks were differentiated by inheriting the neighboring ranks (e.g., a virus having no order but having *Poxviridae* as family, would acquire an order called n_*Poxviridae* to make it different from other viruses unclassified at the order level). Order inherited missing labels from family, whereas family inherited order (if available), and so on. Multiple missing ranks inherited multiple unidentified labels. All permutations were considered and curated as to avoid unwanted duplicity.

Finally, the genome coordinates of each read were used to create an ideal assembly for each dataset, a hypothetical best attainable assembly build by ordering reads into larger contig-like structures for every genome. To achieve this, all reads were first separated by their genome of origin (GI). Then, for each genome all its reads were sorted by its known start position within the reference genome. A contig was formed by overlapping end positions with every new read start. If no overlap was found, the read was the seed to a new contig. This allowed us to calculate the same assembly statistics in order to compare the success of other assemblies with the ideal assembly posing as the goal or target.

### Clustering and ordination methods

In order to compare the different assemblies, a dimensional reduction Principal Component Analysis was performed with in-house R scripts (R programing language ver 3.0.2, 2013-09-25) to limit the number of variables. Only the variables that had a statistically significant association with each of the dimensions obtained by the PCA were kept as the variables that explain the clustering configuration. Hierarchical clustering analyses were performed with R scripts to illustrate closeness to the ideal assemblies. A linear discriminant analysis (LDA) was performed in order to find those variables that best define the clusters. The variables that correlated with the first two axis of the PCA and the ones selected with the LDA were the ones that were used as predictors for the grouping in the hierarchical clustering analysis (Table S5 in Supplementary Material).

### Coverage and fragmentaion spectra

A contig-coverage spectrum was calculated for every assembly with in-house Perl scripts by analyzing the distribution of the reads mapping each contig. Using the resulting coordinates, coverage was assigned. Roughly, this number would be the number of copies of a contig that would be present in a given assembly. Additionally, both species-coverage and species-fragmentation spectra were calculated for each assembly considering all reads that matched a GI (pointing to a single species). The resulting coverage and fragmentation distributions were then used as a proxy of the abundance and completeness of each genome given a certain assembler.

In order to obtain an index combining the effect of the coverage and the fragmentation of a given genome, we generated a Penalized-Coverage index that was obtained as the product of the coverage and the fragmentation (with the latter spanning from 0 to 1). In this way, lower fragmentation index values (that is, more fragmented sequences) resulted in a harsher penalization of the coverage. The coverage, fragmentation, and penalized coverage by species are included in Tables S6–S8 in Supplementary Material, respectively.

### Alpha diversity estimators

Four diversity indexes were considered in this study: the Shannon–Weaver entropy index, the Chao1 and ACE richness estimators, and the expected number of species (specNum). They were calculated using the contig spectrum and species spectrum for each assembly using in-house R scripts. The function “diversity” (library: “vegan”) was used for the Shannon index and is calculated as follows:

$$H=\sum_{\text{i}=1}^{\text{S}}p_{\text{i}}{\text{log}}_{\text{b}}p_{\text{i}}$$

where *p*_i_ is the proportion of species i and *S* the number of species so that the summation is equal to 1 and b is the base of the algorithm (the default was used: natural algorithm) (Hill, 1973).

The Chao1 index was calculated with the function “chao1” from the fossil package. It is calculated according to:

$$S_{\text{Chao1}}=S_{\text{obs}}+\frac{n_{1}^{2}}{2n_{2}}$$

where *S*_obs_ is the number of observed species, *n*_1_ the total number of singletons and *n*_2_ is the number of doubletons (Chao, 1987).

The ACE richness index was calculated with the ACE function from the library fossil. The index is calculated as follows:

$$S_{\text{ACE}}=S_{\text{rest}}+\frac{S_{\text{rare}}}{C_{\text{ACE}}}+\frac{F_{1}}{C_{\text{ACE}}}\gamma_{\text{ACE}}^{2}$$

where *S*_rare_ is the number of species with <10 observations, *S*_rest_ is the number of species with more than 10 observations, *C*_ACE_ is the sample coverage *F*_i_ is the number of species with i individuals, and $\gamma_{\text{ACE}}^{\text{2}}$ is the coefficient of variation, calculated as

$$\gamma_{\text{ACE}}^{\text{2}}=\text{max}\left[\frac{S_{\text{rare}}}{C_{\text{ACE}}}\frac{\sum_{\text{k}=1}^{10}k(k-1)f_{\text{k}}}{\left(N_{\text{rare}})(N_{\text{rare}-1}\right)}-1,0\right]$$

(Hughes et al., 2001). Finally, the total number of species was calculated using the function “specnumber” (library: “vegan”), which gets the number of different items in the sample. Finally, an additional calculation of the Shannon index was calculated from a contig spectrum (a distribution of contigs having n number of reads associated) using the offline Octave implementation of the Phage Communities from Contig Spectrum (PHACCS) script (Felts et al., 2005).

The PHACCS method is based on a modified version of the Lander–Waterman algorithm, generating contig spectra that are fitted to a selected model (power law in our study). The most accurate spectrum is selected by calculating the error. An expected size of the items is inputted, which in the present study was selected as the average length of the genomes.

The genlengths parameter was set as the mean contig length of all included genomes. The contig spectra were calculated using in-house Perl scripts.

The assemblies were compared by performing a dimensional reduction with a Principal Component Analysis. Only the variables that had a statistically significant association with each of the dimensions obtained by the PCA were kept. Correlations of each of the variables to the different components are presented in Table S9 in Supplementary Material.

### Statistical analysis

The statistical significance for all the different comparison performed in the current analysis were carried out by means of the Kruskal–Wallis one-way analysis of variance by ranks implemented in R scripts the corresponding *p*-values were adjusted (reported as *q* values) by multiple testing using the Benjamini and Hochberg correction Correlations were performed using the Spearman correlation.

### Association methods

The assembly statistics provide an insight into the success of genome reconstruction and they can be used to assess the effect on the diversity calculation. In order to determine whether the assembly statistics had an influence on the alpha diversity, we implemented three different association methods: the Spearman correlation index (SCp) and two multivariate approximations: a generalized linear model (GLM), and random forest, an ensemble learning method for regression. The alpha diversity estimators were set as the response variable and the assembly estimator as the predictors. For more technical details, please refer to the additional methods file (Presentation S1 in Supplementary Material).

## Results

### Simulated data

The simulated reads containing 0.05, 0.5, and 5M reads were quality filtered and trimmed, resulting in the elimination of 755, 7669, 76,147 reads, respectively, accounting for 1.5% of the total reads in all cases. Reads were removed both on the forward and reverse sets as no singletons were considered for the rest of the analysis. This resulted in a dataset size of 49,245, 492,331, and 4,923,853. As expected, none of the statistic tests against the real abundance data (the one the reads were generated from) rejected the hypothesis of them having the same distribution. The abundance of the species for the three simulated datasets are shown in Table S1 in Supplementary Material.

### Assembler performance

The assembly statistics for the nine assemblers (CLC, IDBA, MEGAHIT, MIRA, RayMeta, SOAPdenovo, SPAdes, Velvet, and Metavelvet), as well as that from the ideal assembly are shown in Table S2 in Supplementary Material, including the N50, Largest contig, Percentage of chimeras at different taxonomic levels (species, genus, family and order) and GI, Mean Fragmentation and Mean contig coverage, along with the total reads assembled.

The N50 is an estimator of the overall length of the assembly and it increased predictably in the larger datasets. Unsurprisingly, all assemblers managed to build shorter and fewer contigs when using the low sequencing depth 0.05M dataset and the larger and more abundant ones with the 5M, reflecting the effect of sequencing depth. Congruently, the number of chimeras at all taxonomic levels raised from 0.05 to 0.5M as happens when depth is increased because the probability of adding sequences belonging to other species (bad k-mers or alternative paths in the graphs of de Bruijn algorithms) is incremented as well. The increased number of reads in 5M dataset seemed to buffer chimera formation as CLC, MEGAHIT, Meta-IDBA, SOAPdenovo, and Velvet managed to build fewer chimeric contigs when compared to the 0.5M dataset most probably by providing the necessary sequences to close gaps in the genomic sequences. Chimeras in lower taxonomic levels were more abundant as most similarity would be expected in similar species or genus, allowing the formation of spurious structures.

Although coverage varied considerably between datasets, regardless of the assembler, the mean fragmentation reported fewer changes, even remaining virtually unaltered in some cases. This may indicate read recruitment in higher sequence depth sets added up to the existing fragments assembled (coverage) but had a faint effect in closing gaps.

With an average of 58.83 ± 20.03%, the percentage of assembled reads for most assemblers was rather low (as compared with the ideal assemblies which had an average percentage of 97.25 ± 3.82%). Only the SPAdes, Ray Meta, and CLC assemblers used a similar percentage of the reads but only on the 5M dataset, whereas the other assemblers managed to assemble a higher proportion in the 0.5M set (with the exception of MIRA).

### Effect of the assembler

Results were then compared by assembler (using the average and SD of all three datasets in each statistic, Table S3 in Supplementary Material). Although the assemblers were not shown to be statistically different for each of the parameters tested in the current analysis, SPAdes, CLC, and RayMeta managed to build the longest contigs, had fairly big N50 values, had some of the lowest number of contigs and showed the highest mean coverage. However, congruent to what was reported before (Vázquez-Castellanos et al., 2014), the assemblers that manage to maximize the contig lengths and the coverage often present the highest number of chimeras. The 5M SPAdes was particularly populated by chimeric contigs (11.54 ± 3.24%), which seems to result from the assembler using more sequences than most other assemblies (82.96 ± 11.81%).Conversely, the RayMeta and the CLC assemblers managed to assemble most reads into contigs forming larger but fairly chimeric sequences. SPAdes achieved the best level in mean fragmentation (0020.0097 ± 0.0015) but was still far from the ideal assembly equivalent (0.2803 ± 0.2739).

Velvet (developed for genomic assemblies with a single expected coverage peak) produced the lowest percentage of reads assembled (29.69 ± 11.75%), in part explaining why it had the worst value in the average largest contig, the second lowest number of contigs, mean coverage, and the fewest chimeras proportionally. Metavelvet stood out with the largest average N50 (7732.33 ± 10,996.70, although mostly due to the N50 of 20427 in the 5M dataset but not nearly as impressive in the other two sets).

MIRA had the shortest overall assembly size (N50), the highest number of contigs and the worst fragmentation values, meaning it managed to assemble multiple contigs but could not extend them or assemble them together, resulting in the formation of a huge collection of micro-contigs. And the rest of the assemblers managed results between these cases.

### Effect of the sequencing effort

The comparison (Table S4 in Supplementary Material) between datasets showed the assemblies of the 0.05M set obtained in average lower values in every single statistic. The 0.5M set could be seen as an improvement in the sequencing depth resulting in improved mean coverage and reads assembled values with respect to the ones that were observed in the 0.05M dataset. The highest value even reached the highest values for the largest contigs and the percentage of reads assembled. The number of chimeric was also the highest for all the three datasets. The assemblies retrieved from the 5M dataset shown the highest values for the N50, Mean_Fragmentation, and the contig coverage. The percentage of chimeric contigs was lower than that of the 0.5M dataset.

It was interesting, however, that the 5M dataset did not manage to get the highest average percentage of reads assembled in favor of its 0.5M counterpart. As mentioned before, only SPAdes, CLC, RayMeta, and to some extent MIRA managed better results in this department. Perhaps partly as a consequence, the percentage of chimeric contigs was in average higher in the 0.5M dataset. However, a higher sequencing depth is also expected to reduce the gaps between sequenced regions, therefore favoring longer and more accurate contigs with best support (high coverage). The Mean_Fragmentation value was the only variable that proved statistically different, with the 5M dataset maximizing the score (the less overall fragmented ones).

**Figure 1:**
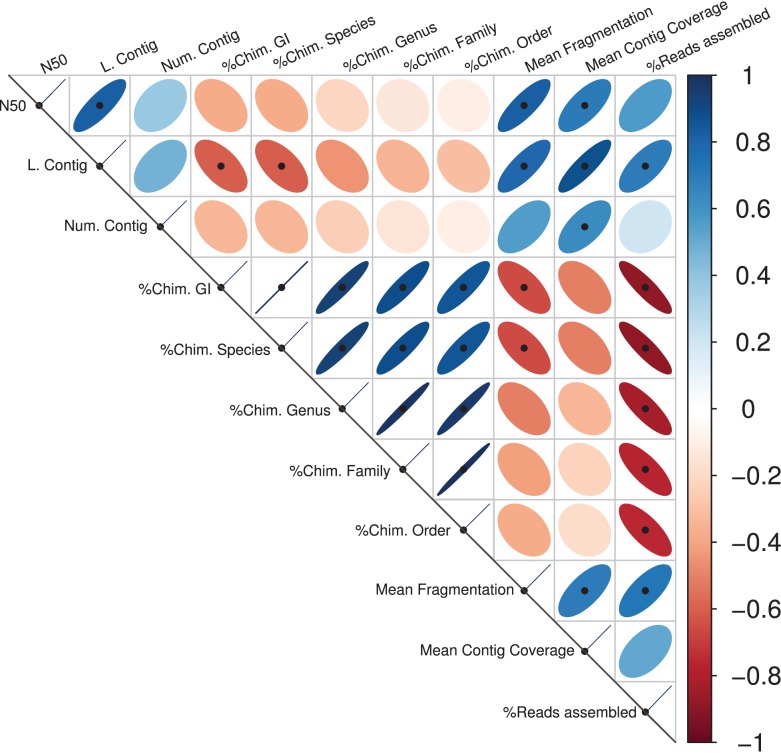
**Assembly statistics correlations**. Spearman’s rank correlation coefficient for all available pairwise combinations of the assembly statistics. The width of the ellipses and the color gradient indicate the strength of the correlation. Blue ellipses tilted right represent positive correlations; negative ones are represented by red ellipses that tilt left. Black points represent statistically significant correlations.

Significant correlations between the different assembly statistics are shown in Figure 1. As expected, the contig length variables (the N50 and the largest contig) values showed positive correlations with the mean contig coverage and with the Mean Fragmentation. Congruently, the percentage of reads assembly correlated positively with the largest contig, the Mean Fragmentation and negatively with all the different estimators of chimeric assemblies. The five different taxonomic levels of chimeric assemblies correlated negatively with the percentage of reads assembled, indicating the limited capacity of an assembler is not only reflected in the low percentage of reads assembled but also in the taxonomic accuracy of the resulting contigs or scaffolds. The Mean Fragmentation per assembly correlates negatively with all the percentage of chimeras of the five different levels in the current analysis, but the correlation to the GI and species level were statistically significant.

### Cluster formation

**Figure 2:**
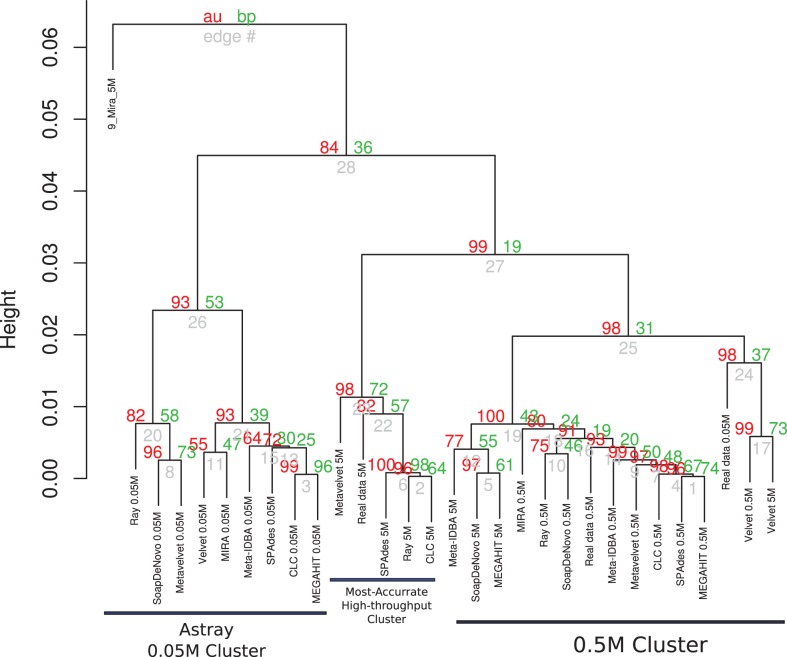
**Hierarchical clustering of assemblies based on performance**. Dendrogram produced with hierarchical clustering calculated from the correlation of the assembly statistics of each assembly and the three datasets. Red numbers represent the Approximately Unbiased *p*-val (AU). Green numbers are the Bootstrap Probability (BP) value. Three clusters were highlighted and supported by AU *p*-val, the Astray 0.05M Cluster, the Most Accurate High-throughput Cluster, and the 0.5M Cluster.

The results of cluster formation of the different assemblies are shown in Figure 2 and Presentation S1 in Supplementary Material. Apparently, sequencing depth may have had a major influence as some clusters of the same datasets may be seen. Namely, all assemblies using the 0.05M dataset were clustered together but they were astray from the corresponding ideal assembly. This may be due to the high variability of the results for this set, as these assemblies are characterized by short low-coverage contigs as well as highly fragmented contigs. A second cluster was formed containing the assemblies of the 0.5M set, along with their ideal assembly for that depth. Except for SPAdes, RayMeta, and CLC, most assemblers had more reads assembled into contigs for this set. Finally, a small cluster of 5M assemblies of SPAdes, RayMeta, CLC, Metavelvet, and the ideal assembly for that set was detected, all of them having relatively few contigs but using most of the reads as supported by coverage.

The PCA in Figure S1 in Supplementary Material supports the clustering effect of the sequencing depth on the behavior of the assemblers. Depending on the total number of pair-end reads, assemblers were ideally closer to their respective ideal assemblies. In order to determine the most important variables to the clustering and we combined the variable selection retrieved from a LDA with those variables that posses a statistically significant association with each of the dimensions of the PCA (Table S4 in Supplementary Material). The cumulative variation explained by the two principal components of the dimensional reduction comprises 91.12% of total variation. The first component of the PCA was mainly influenced by the combined effect of the variables that measures the quality of the assembly (N50, Largest Contig,%reads assembled, Mean contig coverage, and the Number of contigs) and the alpha diversity estimators (Contig specSpecies, Contig ace, Contig chao, GI specSpecies, Gi ace, Contig Shannon, and PHACCS Shannon), while the second component relays mostly in the length and coverage of the assemblies (N50, Mean contig coverage, and Largest contig). The LDA predictions indicate the number of contigs and the alpha diversity estimators derived by the contig counts (Contig specSpecies, Contig_ace, and Contig chao).

The SPAdes, CLC, RayMeta, and Metavelvet 5M assemblies grouped closer to the respective 5M ideal assembly. The utterly fragmented MIRA assemblies showed as outliers in the ordination and the clustering analysis.

### Coverage and fragmentation

An important feature of any assembly software is its capacity to assemble reads accurately into non-chimeric contigs. Greedy assemblers tend to join multiple reads long contigs bearing high coverage values, inevitably incurring in the increase of chimeric intake. On the other hand, strict assemblers tend to produce highly fragmented but accurate clusters, limiting the number of chimeras. In order to assess this contrast, we evaluated the coverage and the fragmentation for each viral genome. Several contigs may match the same genome in each assembly. This coverage and fragmentation profiles were calculated for each of the nine different assemblers used in this study with the three sequencing depths (0.05, 0.5, and 5M datasets). The values were compared with those of the ideal assemblies.

**Figure 3:**
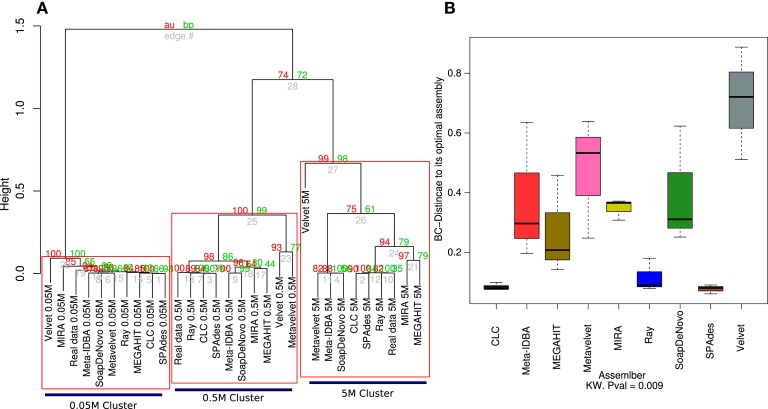
**Hierarchical clustering of the estimated species coverage**. Dendrogram generated from hierarchical clustering calculated from Bray–Curtis distances calculated from the species coverage calculated for each assembler **(A)**. Red numbers represent the Approximately Unbiased *p*-value (AU). Green numbers are the Bootstrap Probability (BP) value. Three clusters were highlighted and supported by AU *p*-values. Boxplots of the Bray–Curtis distance of all three assemblers with respect to its ideal assembly **(B)**. Kruskal–Wallis test *p*-values were calculated for the whole comparison.

The genome coverage profile for all nine assemblers showed that distinct sequencing efforts lead to differences greater than those that are due to the assembler that is employed (Figure 3A). Although the effect of the sequencing depth remains the main source of variation and assembly clustering attractor, the Bray–Curtis distance of each assembly to its ideal is not significantly lower for the three different datasets.

The 0.05M cluster was the most dispersed, as expected, while the 0.5M was the tightest one. The Ray, CLC, and SPAdes assemblers were the most consistent to the ideal assemblies for the 0.5 and 5M datasets; this was further supported by the distance to the ideal contig as both were significantly closer than the rest of the assemblers, with Velvet standing out as the assembler that more poorly calculated the relative abundance of each simulated genome. The 5M group presented three sub-clusters: RayMeta, CLC, and SPAdes clustered together with the Ideal assembly, followed by MIRA and MAGAHIT. The rest clustered together in a separate sub cluster (Figure 3A). This is similar to the feature comparison in which CLC is closer to SPAdes and next to Ray, with the largest percentage of reads assembled and the rest had similar values (~52%). The rest appear further, with the highly variable MEGAHIT as the closest (Figure 3B).

**Figure 4:**
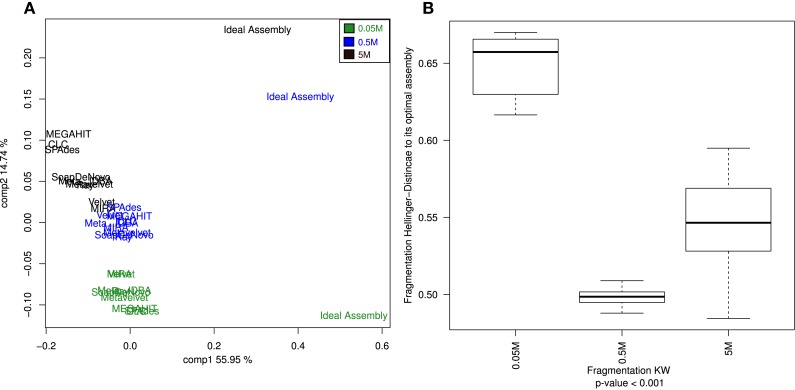
**Principal Coordinates Analysis (PCoA) of the estimated genome fragmentation**. PCoA of the Hellinger distances calculated from the fragmentation of each genome given an assembly **(A)**. Assemblies of the 0.05M dataset are in green, in blue the ones assembled from the 0.5M, and in black the ones from the 5M set Boxplots of the Hellinger distances of each of the three dataset assemblies (0.05, 0.5, and 5M) with respect to their corresponding ideal assemblies **(B)**. Kruskal–Wallis test *p*-values were calculated for the whole comparison.

The fragmentation in all assembling efforts fell far from the actual expected values (Table S2 in Supplementary Material) of the ideal assembly. This is reflected by the clustering profile, in which the ideal fragmentation clustered separately, far from the rest of the assemblies (Figure 4A). Again, the sequencing depth was shown to be an important factor (statistically significant) in order to improve the fragmentation values, even more influential than the assembler of choice (Figure 4B).

Finally, a penalized-coverage index was calculated as the product of the coverage and the fragmentation of each genome using the latter as a penalty factor to obtain a combined deflated value, an adjusted estimator of each genome completeness in the assembly (Table S7 in Supplementary Material). The effect of the coverage is constrained by the fragmentation as having a larger coverage does not imply genome completeness.

The clustering analysis of the penalized-coverage values for the 5M dataset assemblies showed that those made with CLC, SPAdes, Ray, and MIRA were more similar to all three ideal assemblies than the rest (Figure S2 in Supplementary Material). Sequencing depth seems determinant in the success of assembly of large contigs, which can be considered longer fragments of the corresponding genome (bearing fewer gaps). As the depth is increased, the contig distribution is expected to resemble the species distribution more closely, provided the number of chimeric contigs remains negligible.

### Diversity in contigs and species

Four diversity indexes were used to estimate alpha diversity of each assembly: Shannon’s index that analyzes entropy in a sample, Chao1 richness estimator, which magnifies singleton contribution, ACE, a richness estimator considering the items occurring from 1 through 10 times, and expected species estimator. All four were calculated with the different contig composition and with the GI composition (contigs are counted separately whereas GIs account for one each). Finally, a Shannon index was calculated using PHACCS (see Materials and Methods).

**Figure 5:**
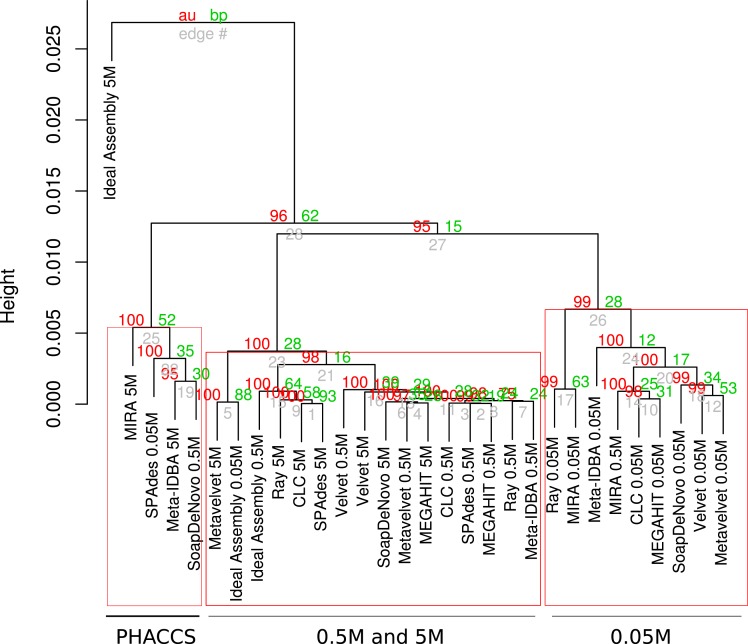
**Hierarchical clustering of assemblies based on its alpha diversity estimators**. Dendogram build from hierarchical clustering calculated from the correlation calculated from the Alpha diversity statistics estimators for each assembly. Red numbers represent the Approximately Unbiased *p*-value (AU). Green numbers are the Bootstrap Probability (BP) value. Three clusters were highlighted and supported by AU *p*-values: The PHACCS clusters, the 0.5 and 5M cluster and the 0.05M cluster.

The assemblies produced in the current analysis showed three different clusters based on the estimation of their alpha diversity statistics. This configuration is mostly driven by the extreme values of the very fragmented MIRA_5M, with the highest number of total contigs assembled (short but very abundant) assembly and high PHACSS values (Figure 5).

The clustering of all assemblies resulted in the formation of three groups, clearly defined by the sequencing effort as reflected by the three different datasets, regardless of the assembly (Figure 5; Figure S3 in Supplementary Material). The 0.5 and 5M cluster showed that the 5M Metavelvet assembly and the CLC, SPAdes, and RayMeta assemblies were the ones that clustered closer to the 0.05 and 0.5M datasets, indicating that the fragmentation and the percentage of reads assembled of such assemblies are similar to the ones observed on the shorter datasets, reflecting such behavior in the estimation of the alpha diversity estimators. All assemblies were clearly off from the 5M Ideal Assembly. The 0.05M cluster were formed by those with the lowest values for the alpha diversity (Figure 5), this mainly due to the fact that having low percentage of reads assembled leads to an incomplete reconstruction of the sampled community.

The cumulative variation explained by the two principal components of the analysis sums 95.92% of the total variance. The first principal component proved to be a very influential factor explaining the 90.16% of the variation (Figure S3 in Supplementary Material) and was influenced by contributions of most alpha diversity estimators (Table S6 in Supplementary Material). The second component just explains the 5.76% of the variation and it just influenced by the Shannon diversity index (Table S6 in Supplementary Material): this second component is the one that separates the 5M Ideal assembly and the 5M MIRA assembly. The rest of the assemblies seem to be distributed across the first component.

The alpha estimators yielded very different values, depending on whether contigs or species were used to predict the diversity. Three calculations of alpha diversity estimators were used for each assembly: by contig, by GI, and by PHACCS. The first two, when analyzed by contig or by GI spectra, were virtually equal. Analyzing by contig showed lesser sources of variation given the assembly when analyzed by PHACCS, with no congruence between the three association methods.

**Figure 6:**
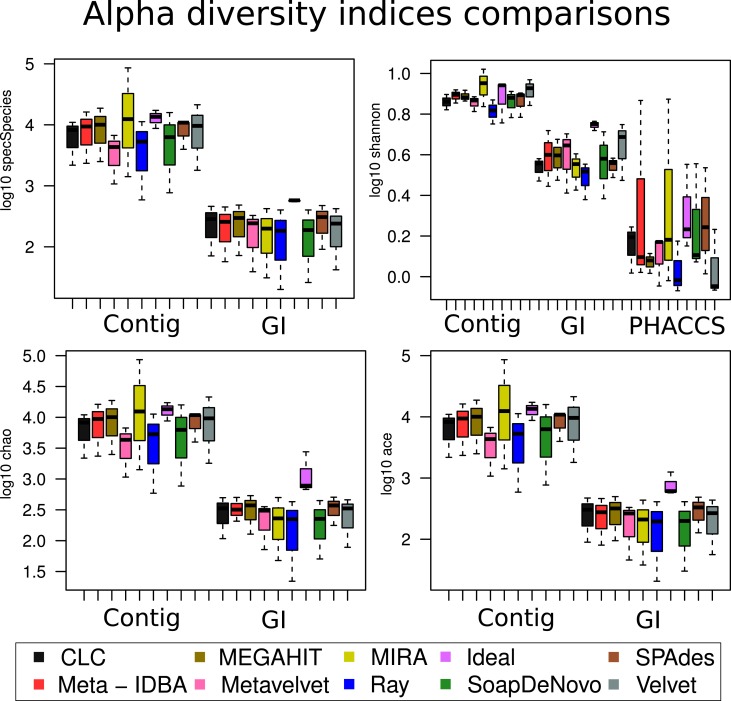
**Alpha diversity index comparisons**. Boxplot of the distribution of the estimation of all the different alpha diversity metrics used in the analysis with the three different types of estimations: The species-coverage estimation (GI), the one based on contig spectrum and the PHACCS estimations using the total number of contigs.

When the different assemblers were compared (calculating the mean value for all three datasets for each assembler), no difference was significant within the same method (calculations with contig, with GI or PHACCS) but was actually different between the groups (Figure 6): regardless of which diversity index was estimated, whenever diversity was calculated from contigs, the index was overestimated. This was because the higher number of different contigs that could be used as different species was higher than total different GIs. Diversity calculated from GIs was more accurate as one GI always corresponds to a single species. PHACC’s Shannon index was underestimated in all cases, with a larger variability than any of the other methods or indexes. This was due to the way species are calculated by PHACCS.

### Relation of the diversity and assembler parameters

In order to determine which assembler statistics may have an influence on the Alpha diversity, the Spearman correlation index, the GLM using LASSO and Random Forest were implemented.

**Figure 7:**
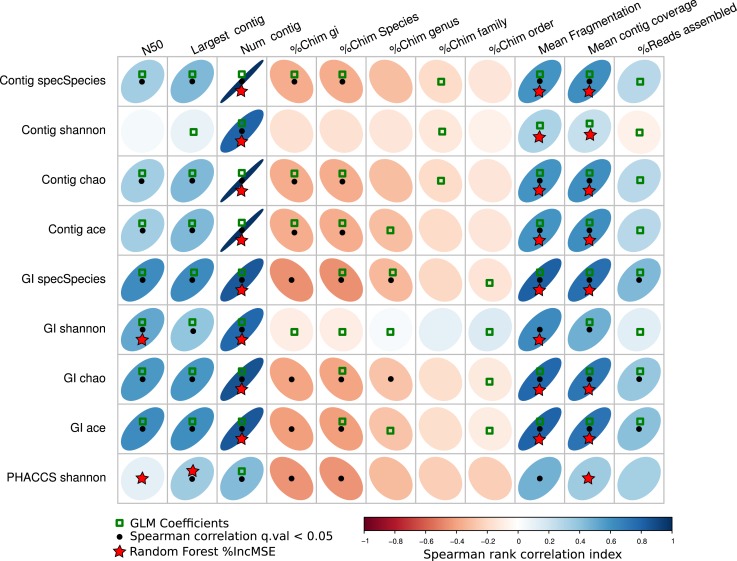
**Associations of the assembly statistics and the alpha diversity metrics**. Correlation plot representing all possible associations between the assembly estimators and the alpha diversity estimators. Black dots represent Spearman’s rank correlation with statistical significance. Green boxes represents all the assembly statistics that were significant for the generalized linear model that predicted the alpha diversity index, the red stars represent variables that most increase the mean square error (MSE) for the model determined by a random-Forest algorithm. Blue ellipses tilted right represent positive correlations, whereas negative correlations are represented by red ellipses tilted left. Black points represent statistically significant correlations (*q*-value <0.05).

Figure 7 resumes the associations predicted by the three regressions methods. The GLM method predicted more associations between the diversity estimators than the other two, suggesting most parameters may have an influence on the diversity. The other two, the percentage of increment of the mean square error (%IncMSE) produced by Random Forest, and the significance Spearman correlation *p*-values (SCp), were more conservative in establishing associations between the alpha diversity estimators and assembly parameters, avoiding most of the chimeric contigs.

Most importantly, the SCp coefficients predicted that length of the contigs (Largest contig and N50 estimators), the fragmentation of the assembly and associated estimators (Number of contigs, Mean Fragmentation, and Mean contig Coverage) may actually influence modifications of the alpha diversity estimators.

Finally, the %IncMSE was the most conservative across the different diversity estimators, only supporting the role of the Fragmentation of the assembly and related estimators (Number of contigs, Mean Fragmentation, and Mean contig Coverage) as the major source of variation of the alpha diversity.

When comparing the joint prediction for the three methods, we determined that the mean fragmentation of the assembly, the mean contig coverage, and the number of contigs are the most likely to have an influence on the alpha diversity as they are the best supported.

## Discussion

Current technologies are aided by the use of assemblers to better assign taxonomic and functional annotations to the sequences in samples. As we have seen in our analysis, the success of assembling is dependent on different parameters, mainly the sequencing depth and the assembly of choice. Our datasets were drawn from actual viral metagenomic by Reyes et al. and are limited by the extend of this dataset. Future studies with different datasets (thus with different abundances) will help further support the results.

Available assemblers yield highly variable results but can be generally divided into two groups: those assembling large contigs with not only high coverage and a high percentage of reads assembled but also a high number of chimeric content at the species level and another group composed by assemblers that manage to assemble considerably less contigs but have fewer chimeras (namely Metavelvet). Predictably, the latter ended up forming shorter contigs and coverage values. On the bright side, these are less chimeric. This was similar to results obtained in other studies (Aguirre de Cárcer et al., 2014).

Another important observation is that no assembler managed to produce contig spectra that accurately emulate that of the best possible assembly. This was supported by the comparisons against the ideal assemblies in which it can be seen that. However, the SPAdes, CLC, and RayMeta assemblers fared acceptably, posing as the best options for viral metagenomic assembling. This is largely due to the fact that most genomes fail to assemble around half of the total sequences in average whereas the SPAdes, CLC, and RayMeta end up forming a fairly large number of chimeric sequences.

Even though the increase of sequencing depth was reflected by a global increment in the percentage of reads assembled (which was evident in a step from the 0.05M to the 0.5M set), this was not the case for all the assemblers in the step from the 0.5M dataset to 5M. In fact, only SPAdes, RayMeta, and CLC actually managed to secure an increase in the percentage of reads assemblers (also portrayed by the ordination methods). This was also supported by the penalized-coverage score, which position SPAdes, RayMeta, and CLC closer to their corresponding ideal assemblies.

MIRA barely saw any changes in the percentage and the rest (SOAPdenovo, MEGAHIT Meta-IDBA, Velvet, and Metavelvet) actually achieved self-greater percentages in the 0.5M datasets. Meta-IDBA resulted the most accurate (when compared to real data) with this dataset. This difference may be due to the inner workings of their algorithms and suggest that they do not cope as well with the increased complexity of the deeper metagenomes, perhaps because of the difficulty to resolve correct paths in the de Bruijn graphs. Overall, the 0.5M dataset resulted in the most congruent viable results between assemblers (the cluster for the 0.05M was tighter but also even farther to that of the ideal assembly). In general, it seems that genome assemblers are not well suited for metagenome assembly which is congruent to previous analyses (Smits et al., 2014; Vázquez-Castellanos et al., 2014).

The divergence from the ideal assemblies was highlighted by diversity estimators calculated for each assembler. When hierarchically clustered, the most accurate 5M assemblies (CLC, SPAdes, RayMeta, and Metavelvet) were grouped with the 0.05 and 0.5M ideal assemblies. The 5M fell within the same cluster but as the outer branch, and still far from any of these. This may be due to the ideal assembly forming several rather complete genomes, deflating the alpha indexes when compared to actual assemblies. There was also a clear effect of the type of data that was selected. Selecting GIs instead of the contigs as input for the calculations would result in what seemed as an overestimated diversity, independent of the assembler or the coverage, whereas PHACCS produced lower alpha diversity values, probably underestimating some. This latter was also highly variable between assemblers.

The mean was length of the genomes was selected for the spectra generated by PHACCS. Although the mean is susceptible to extreme values, trials with the less biased estimators mode and median resulted in equivalent results, as would be expected for samples drawn from a normal distribution.

Our approach gave us an insight into how an assembly of unknown data is carried out and evaluated on different depth scenarios and calculating diversity for each. Whenever the population is unknown, the alpha diversity of the contigs-spectrum without adjustment or taxonomic grouping would be expected to be significantly larger than estimations using the known species. Thus, it is always advisable to try to classify sequences first prior to alpha diversity calculation.

The alpha diversity is also heavily influenced by the fragmentation and the total number of contigs. MIRA, for example, has far more contigs than the rest of the assemblies using the 5M dataset. This results in the formation of several micro-contigs and clearly overestimates diversity. Thus, the fragment completeness pretty much dictates the accuracy of alpha diversity.

The correlation analysis and association methods (A random-forest algorithm and GLM) suggested the number of contigs, the coverage and the fragmentation score as the assembly parameters that had a greater influence on the diversity. Dataset fragmentation seemed to have broken down what should have been single long contigs into smaller ones, resulting in a higher diversity (a single species is detected as several as the contigs do not necessarily represent species). Chimeric contigs were not really affecting the diversity analyzes. Therefore, the selection of the assembler is critical to the estimation of alpha diversity. It is advisable to use assembler that minimize fragmentation and increase coverage, while keeping the number of contigs within a reasonable range.

This study summarizes the effect of the coverage and fragmentation of genomic sequences in the use of different assemblers and the calculation of the alpha diversity. The main limitation of the alpha diversity estimation is the highly fragmented viral genomes into that result into several small and low-coverage contigs. The fragmentation is first driven by the sequencing effort and in second by the algorithm used to deal with metagenomic data for genomic assembly. Assemblers that tend to minimize the number of contigs and maximize the length indicators such as the N50 and the percentage of assembled reads fare better in obtaining more reliable estimates of alpha diversity. The assemblers SPAdes, CLC, and Ray-meta comply with these specifications, clustering closer to the ideal assemblies when comparing the assembly statistics, the alpha diversity estimators and the species profiles.

The fragmentation is an intrinsic problem of viral metagenomes, even with the ideal assembly of higher sequencing depth. Thus, a large number of viral genomes remain fragmented in small contigs even with high-throughput sequencing. Furthermore, with high percentages of unknown sequences in viral metagenomes, alpha diversity calculated from contigs is expected to be an overestimation since most fragments will remain unassembled. Assemblers cannot cope with these challenges alone and require different strategies combined with binning software to minimize both problems.

## Author Contributions

AM, JV-C, and RG-L conceived the experimental design. JV-C and RG-L performed the computational analysis, interpreted and discussed the results, and wrote the draft of the manuscript. AM provided funding and the computational resources. All authors read and approved the final manuscript.

## Conflict of Interest Statement

The authors declare that the research was conducted in the absence of any commercial or financial relationships that could be construed as a potential conflict of interest.

## Supplementary Material

The Supplementary Material for this article can be found online at http://journal.frontiersin.org/article/10.3389/fbioe.2015.00141

## Funding

This research was supported by grant SAF-2012-31187 from the Ministry of Economy and Competitiveness (MINECO), Spain to AM. RG-L was recipient of the CONACYT-CECTI fellowship, Mexico. JV-C was recipient of a CONACYT-SECITI fellowship, Mexico.
