# Supplementary material for: Fragmentation and Coverage Variation in Viral Metagenome Assemblies, and Their Effect in Diversity Calculations
Source: Front Bioeng Biotechnol. 2015 Sep 17;3:141. doi: 10.3389/fbioe.2015.00141 (PMC4585024; doi:10.3389/fbioe.2015.00141)
Supplement: Supplementary file 2 [file Table_2.DOCX]

Supplementary Table 2: Assembly statistics.

| **Assembler** | **N50** | **Largest contig** | **Num contig** | **%Chim gi** | **%Chim_Species** | **%Chim genus** | **%Chim family** | **%Chim order** | **Mean Fragmentation** | **Mean contig coverage** | **%reads assembled** |
| --- | --- | --- | --- | --- | --- | --- | --- | --- | --- | --- | --- |
| RayMeta 0.05M | 887 | 44627 | 898 | 2.727 | 2.727 | 0.091 | 0.065 | 0.065 | 0.0070 | 9.474 | 38.725 |
| RayMeta 0.5M | 1367 | 137891 | 7154 | 6.229 | 6.214 | 1.725 | 1.551 | 1.500 | 0.0073 | 27.441 | 73.408 |
| RayMeta 5M | 7893 | 244764 | 13900 | 11.374 | 11.329 | 1.768 | 1.326 | 1.266 | 0.0091 | 192.951 | 90.803 |
| Meta-IDBA 0.05M | 696 | 17812 | 2688 | 3.812 | 3.812 | 1.359 | 1.326 | 1.292 | 0.0071 | 2.167 | 50.094 |
| Meta-IDBA 0.5M | 1247 | 53698 | 10664 | 6.103 | 6.036 | 1.342 | 1.087 | 0.994 | 0.0079 | 11.600 | 70.168 |
| Meta-IDBA 5M | 2734 | 145120 | 17837 | 4.227 | 4.180 | 0.887 | 0.637 | 0.567 | 0.0086 | 43.042 | 51.204 |
| SOAPdenovo 0.05M | 720 | 36316 | 1113 | 1.519 | 1.519 | 0.355 | 0.341 | 0.341 | 0.0070 | 7.282 | 38.286 |
| SOAPdenovo 0.5M | 1092 | 137891 | 8132 | 4.566 | 4.553 | 0.981 | 0.854 | 0.833 | 0.0073 | 16.462 | 65.784 |
| SOAPdenovo 5M | 2516 | 244765 | 18935 | 3.501 | 3.471 | 0.630 | 0.475 | 0.408 | 0.0091 | 48.124 | 51.760 |
| CLC 0.05M | 1096 | 44837 | 2274 | 6.841 | 6.829 | 1.647 | 1.464 | 1.375 | 0.0075 | 3.242 | 63.133 |
| CLC 0.5M | 2096 | 137925 | 8683 | 9.931 | 9.876 | 1.685 | 1.375 | 1.205 | 0.0095 | 19.295 | 84.180 |
| CLC 5M | 8017 | 244788 | 11516 | 7.361 | 7.303 | 1.616 | 1.143 | 1.049 | 0.0115 | 166.760 | 90.380 |
| Velvet 0.05M | 668 | 19291 | 2391 | 1.854 | 1.854 | 0.585 | 0.548 | 0.538 | 0.0068 | 1.450 | 37.636 |
| Velvet 0.5M | 925 | 17002 | 12089 | 2.438 | 2.434 | 0.497 | 0.434 | 0.418 | 0.0074 | 3.871 | 35.237 |
| Velvet 5M | 1250 | 33593 | 24524 | 0.676 | 0.644 | 0.135 | 0.081 | 0.057 | 0.0073 | 11.999 | 16.195 |
| Metavelvet 0.05M | 1137 | 44879 | 1328 | 2.626 | 2.626 | 0.920 | 0.849 | 0.788 | 0.0070 | 4.751 | 46.931 |
| Metavelvet 0.5M | 1633 | 102262 | 7893 | 4.242 | 4.232 | 0.891 | 0.766 | 0.742 | 0.0081 | 8.571 | 57.216 |
| Metavelvet 5M | 20427 | 212475 | 4458 | 4.357 | 4.348 | 0.836 | 0.769 | 0.702 | 0.0088 | 63.721 | 50.150 |
| MIRA 0.05M | 602 | 18814 | 2349 | 3.385 | 3.385 | 0.729 | 0.694 | 0.690 | 0.0068 | 3.980 | 31.181 |
| MIRA 0.5M | 744 | 137711 | 23570 | 6.301 | 6.271 | 1.420 | 1.157 | 1.072 | 0.0072 | 15.985 | 48.348 |
| MIRA 5M | 505 | 96753 | 172081 | 9.079 | 9.030 | 2.401 | 1.600 | 1.390 | 0.0074 | 89.415 | 52.535 |
| SPAdes 0.05M | 804 | 44891 | 4217 | 7.796 | 7.777 | 1.862 | 1.671 | 1.499 | 0.008 | 2.182 | 69.491 |
| SPAdes 0.5M | 2016 | 137917 | 11552 | 13.473 | 13.402 | 3.163 | 2.268 | 1.896 | 0.010 | 18.171 | 87.885 |
| SPAdes 5M | 13338 | 244784 | 11624 | 13.354 | 13.293 | 5.004 | 4.041 | 3.019 | 0.011 | 168.231 | 91.512 |
| MEGAHIT 0.05M | 888 | 44861 | 2834 | 5.568 | 5.556 | 1.519 | 1.348 | 1.273 | 0.007 | 2.940 | 60.613 |
| MEGAHIT 0.5M | 1621 | 134380 | 11561 | 8.192 | 8.175 | 1.778 | 1.476 | 1.345 | 0.010 | 14.572 | 78.078 |
| MEGAHIT 5M | 3463 | 211812 | 23310 | 5.775 | 5.712 | 1.243 | 1.015 | 0.900 | 0.011 | 70.799 | 57.544 |
| Ideal Assembly 0.05M | 701 | 48354 | 8728 | 0 | 0 | 0 | 0 | 0 | 0.0373 | 1.858 | 92.877 |
| Ideal Assembly 0.5M | 2273 | 137897 | 17268 | 0 | 0 | 0 | 0 | 0 | 0.2265 | 18.802 | 98.937 |
| Ideal Assembly 5M | 29775 | 368365 | 13512 | 0 | 0 | 0 | 0 | 0 | 0.5771 | 188.390 | 99.945 |
