# Supplementary material for: Fragmentation and Coverage Variation in Viral Metagenome Assemblies, and Their Effect in Diversity Calculations
Source: Front Bioeng Biotechnol. 2015 Sep 17;3:141. doi: 10.3389/fbioe.2015.00141 (PMC4585024; doi:10.3389/fbioe.2015.00141)
Supplement: Supplementary file 10 [file Presentation_1.PDF]

Data simulation

Error model generation was carried out with DRISEE (drisee.py --percent -n 3 -b 10000 -x 10000) using a custom Illumina MiSeq real dataset. The adaptor sequence CTGTCTCTTATACACATCTGACGCTGCCGACGA was previously removed with cutadapt.

Some changes were made in the scripts to make them work with our data. In a first step, BEAR generated the desired number of paired-end fasta files containing no quality information. Reads were set to have come from 500bp fragments with 100 standard deviation (python generate_reads.py -t 1000000 -l 300 -i 500 -s 100). Next, BEAR introduced errors based on quality data taken from the DRISEE generated error model and trimmed reads to 300bp. Headers were corrected using in-house scripts to reflect actual headers in a run, as well as information of the reference genome that was used, the starting and ending positions within such genome of both sequences in a pair and the overlap length (if any).

Three sets were generated containing 5 million, 500,000, 50,000 reads respectively.

Low quality bases were trimmed with PRINSEQ (-trim_left 5 -trim_right 15 -min_qual_mean 25 -ns_max_n 3 -trim_qual_left 35 -trim_qual_right 35 -trim_qual_type mean -trim_qual_rulelt -trim_qual_window 5 -trim_qual_step 1) and problematic sequences were removed (-noniupac -lc_method entropy -lc_threshold 75 -min_len 50).

Clustering analyses and ordination methods

The input matrices, retrieved for the different assembly statistics and all the alpha diversity stimulators for each of the assemblies,, were normalized using the Log10 transformation.

All Principal Component Analyses were performed with in-house R scripts using the “princomp” function (library: “stats”); variables that had a statistically significant association (Spearman correlation p value <0.05) (SSAV) with each of the dimensions obtained with the PCA were kept. These were determined using the “dimdesc” function (library: “FactoMineR”). All the hierarchical clustering analyses were created using the function pvclust (library: “pvclust” function: “pvclust”). Each clustering was supported by 1000 bootstrap iterations. Clusters with over 95% of support were plotted using the function pvrect (library “pvclust” function “pvrect”).

A Linear Discriminant Analysis (LDA) was performed in order to find those variable that best discriminate the cluster determined by the hierarchical clustering analysis using the “sda.ranking” function (library: “sda”), those variables with local False Discover Rate < 0.8 were kept. The variables that were predicted into the LDA and those predicted by the PCA the SSAV were used to evaluate the data association.

The Principal Coordiantes Analyses (PCoA) were performed using the “dudi.pco” function (library: “ade4”) using the the Bray-Curtis distance for the species genome coverage file and the Hellinger distance (both from library: “vegan” function: “vegdist”) for the Fragmentation and the Penalized-Coverage index.

The PHACCS script was run with the following parameters: genlengths = 2,479.85 bp, scenarios = “exponential”, min_g&max_g = 1 to 1000.

Statistical Analyses

The statistical significance for all the different comparison performed in the current analysis were carried out by means of the Kruskal–Wallis one-way analysis of variance by ranks implemented in R scripts (library “stats” function “kruskal.test”), the corresponding p-values were adjusted (reported as q values) by multiple testing using the Benjamini& Hochberg correction (library “stats” function “p.adjust”).

Data representation were plotted using the “boxplot”function (library “stats”) and the coreplot function (library “coreplot”), implemented with the R statistical programming language v. 3.0.2. Correlations were performed using the Spearman correlation (function: “cor.test”). The representation of the correlations was performed using the R package corrplot (function: “corrplot”).

Association Methods

The Spearman rank correlation index between the assembly statistics and the alpha diversity estimators were performed using the R language core package (function: “cor.test”); the corresponding p-values were corrected by means of the Benjamini& Hochberg method (function “p.adjust”). Spearman correlation p-values (SCp) were used for the analyses.

The GLMs were generated using the Least Absolute Shrinkage and Selection Operator (LASSO) (library: “glment” function: “cv.glmnet”) via penalized maximum likelihood. The assembly statistics were set as predictors and each of the Alpha diversity estimators were set as the response variable . All the models were validated by means of n-fold cross validation in order to avoid over-fitting problems.

The Random Forest algorithm was implemented using the R Package randomForest (function: “randomForest”). Predictors and response were selected the same as for GLM. The number of trees used for each model was 1000 and four variables randomly sampled as candidates at each split. For each model we took the top three predictors that maximized the percentage of the increment of the mean square error (%IncMSE).
