# Supplementary material for: Fragmentation and Coverage Variation in Viral Metagenome Assemblies, and Their Effect in Diversity Calculations
Source: Front Bioeng Biotechnol. 2015 Sep 17;3:141. doi: 10.3389/fbioe.2015.00141 (PMC4585024; doi:10.3389/fbioe.2015.00141)
Supplement: Supplementary file 11 [file Image_1.PDF]

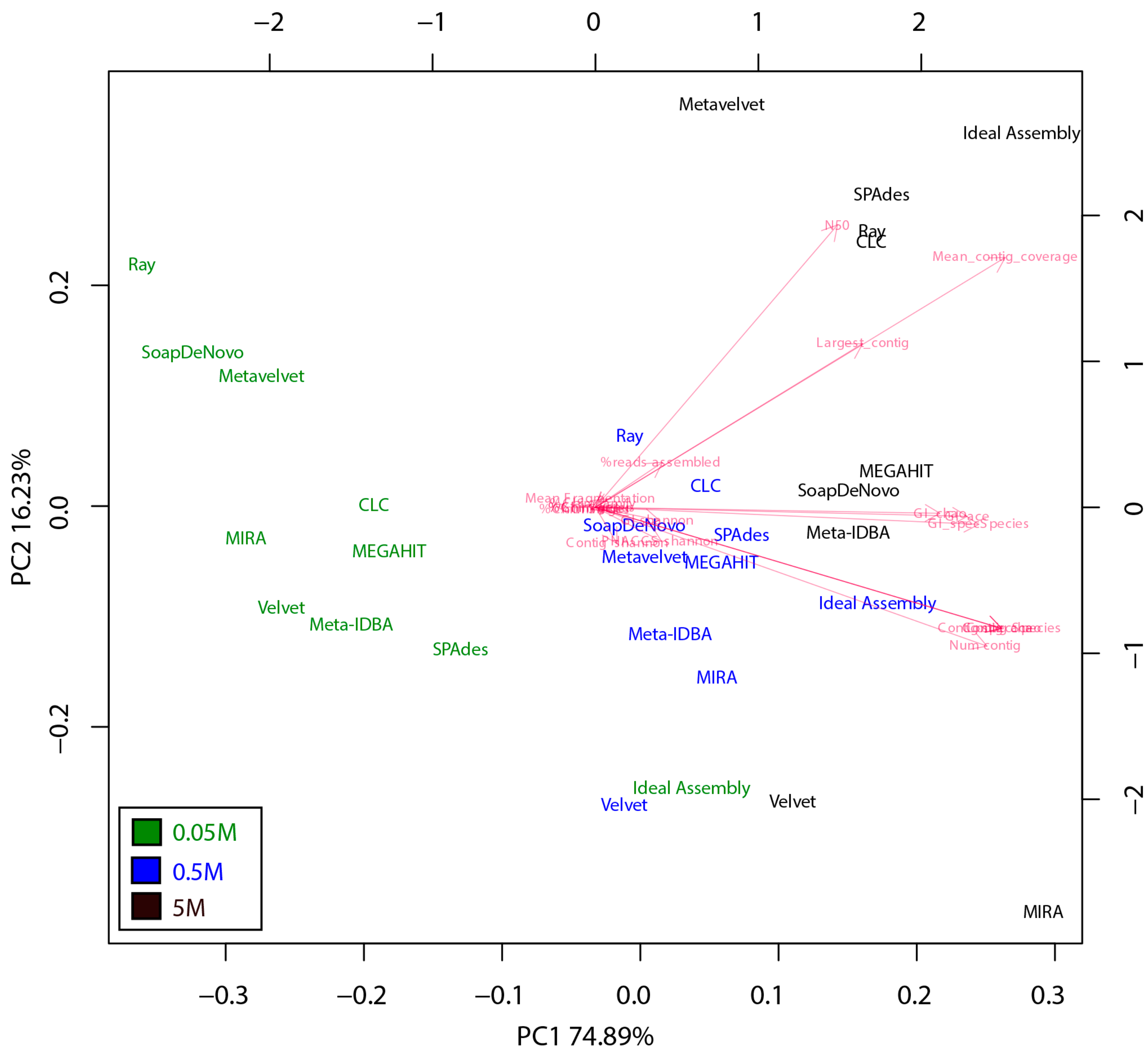


**Supplementary Figure 1 | Principal Components Analysis (PCA) based on the assembly performance.** The two principal components that explain the greatest variation between assemblies are shown for the three datasets; the PCA was produced with the correlation matrix of all the assembly statistics. The assemblies are colored based on the sequencing depth of the input: in green the assemblies from the 0.05M dataset, in blue the ones of the 0.5M and in black the 5M dataset. The length and direction of the arrows represent the coordinates of the assembly-quality variable plotted into the new set of reduced dimensions, the ones established by the two principal components. The length of the vector is proportional to the effect on the component; The orthogonality of the vector is inversely proportional to the effect on the component. A parallel vector would imply a exclusive effect on a given component.
