# Supplementary material for: Fragmentation and Coverage Variation in Viral Metagenome Assemblies, and Their Effect in Diversity Calculations
Source: Front Bioeng Biotechnol. 2015 Sep 17;3:141. doi: 10.3389/fbioe.2015.00141 (PMC4585024; doi:10.3389/fbioe.2015.00141)
Supplement: Supplementary file 12 [file Image_2.PDF]

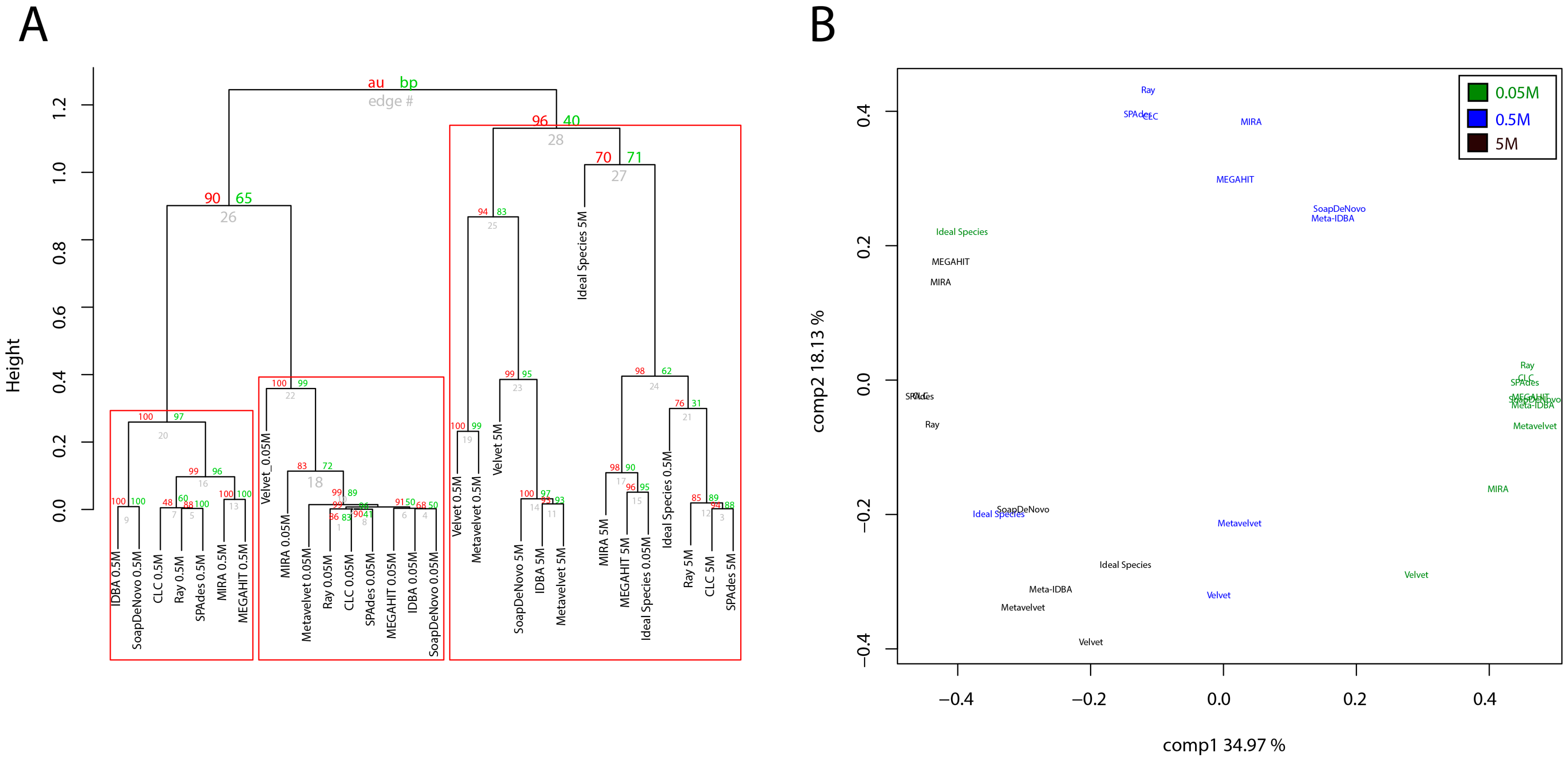

Supplementary Figure 2 | Penalized-Fragmentation Clustering. The hierarchical clustering of the Bray-Curtis distance from the penalized coverage (A) Red numbers represent the Approximately Unbiased p-value (AU). Green numbers are the Bootstrap Probability (BP) value. Three clusters were highlighted and supported by AU p-values and were highlighted by red squares. PCoA of the Bray-Curtis distances calculated from the fragmentation of each genome given an assembly (B).
